# How to Cure Puerperal Eclampsia

**Published:** 1895-08

**Authors:** Emory Lanphear

**Affiliations:** St. Louis, Mo., Professor of Orthopedic and Clinical Surgery in the Woman’s Medical College of St. Louis


For Texas Medical Journal.

### HOW TO CURE PUERPERAL ECLAMPSIA.

BY EMORY LANPHEAR, M. D., PH. D., ST. LOUIS, MO.,
Professor of Orthopedic and Clinical Surgery in the Woman's Medical College of St. Louis.

IN THE beginning of my remarks I will say, I am not an obstetrician—nothing but a plain, every-day surgeon; but I have seen in consultation a considerable number of cases of puerperal convulsions, and know how to cure the disease—which can not be said of all the obstetricians, if I may judge by most of the recent contributions upon the subject: merely repetitions of the same old story of "examine the urine of the patients many days before labor, and if albuminuria is found, cure it"; "bleed"; "use chloral hydrate, or chloroform, or ether, abundantly," etc. If the writers would tell the truth, most of them would confess they never examined the urine of a half dozen pregnant women in all their practice, except in hospital work; women do not go carrying bottles of urine around very often, and the first intimation most patients give of expected confinement, is a message to the doctor to "hurry, or you will be too late." So the first advice amounts to much—theoretically; but to very little, indeed, practically. And as to the second: If those who give the advice so freely would be equally free in relating their death-rates, the details would be far more useful, if less satisfactory to the relator.

But can life be saved in puerperal eclampsia? It can if the patient be seen within a short time after the onset of the spasms. How? By the following methods:

(*a*) *In puerperal convulsions occurring prior to delivery*, the first rule is to chloroform the patient; second, to send for an assistant, if possible to get one quickly; if not, let the husband, or some one else, give the chloroform under close watching, as extreme haste is necessary; third, to empty the uterus *at once;* fourth, to put in practice the method presently to be described, if the seizures return after delivery.

The prime object is immediate delivery of the fœtus—all authorities agree upon this. But the method of so doing is not given anywhere to my satisfaction. The practice I follow is this: If the os be dilating and dilatable, I rapidly enlarge the opening until the long forceps can be applied to the engaging head, or the hand can be introduced to perform version and speedy delivery. In many cases this can be done inside of a half hour. If not, then the proper thing to do is to take the scissors and cut the cervix freely upon each side, clear up to the cervico-vaginal junction, thus producing an artificial, double laceration of the cervix uteri. This will give plenty of room for the passage of the forceps and the child. The only precaution to be observed is to be certain not to cut through the vaginal wall. If the outlet be very close, as in primiparæ, I do not hesitate an instant about making a clean cut through the perinæum also, but not through the muscle near the anus. As soon as the placenta is removed, the os is to be caught and pulled down so as to allow six or eight catgut stitches to be inserted in the cuts in the cervix. The perinæum, if injured, is sewed up, irrigation practiced, and then anæsthesia discontinued. Generally this will be all that is needed.

If there be any abnormality of the pelvis, if the os be so high and so contracted as to render the operation, I have just described, impracticable—as may possibly occur sometimes—Cæsarian section is justifiable if a surgeon who understands the technique is obtainable. The operation I prefer will be described in full in an early number of *Medicine*, the new journal published in Chicago. The entire work ought to be done inside of thirty or thirty-five minutes—the same limit of time I have given for delivery by forceps, the limit of safety. CRANIOTOMY UPON THE LIVING CHILD IS NEVER JUSTIFIABLE; it is murder of an innocent child whose life might be preserved. In the hands of even moderately skillful surgeons, the Cæsarian or the Porro operation is safer for the mother than is craniotomy. And in these days skillful surgeons can be found beside almost every babbling brook—certainly within a short distance, except in the most thinly-settled portions of the country.

After delivery, some simple remedy to excite the activity of kidney and tranquilize the nervous system, will be all that is needed.

(*b*) *In puerperal convulsions occurring after delivery*, whether effected naturally or artificially, we have to deal with a more serious problem, but one easily solved by the following plan of treatment: Open a vein and inject from one pint to one quart of normal salt solution! This dilutes the toxines which cause the convulsions, and increases the arterial tension to such an extent as to restore urine secretion even if there be total suppression; within a half hour, urine will be found in a bladder previously empty, in a vast majority of cases. In a few bad cases, it will not have this effect, in which instances the spasms will return in an hour or so. If they do, another intra-venous injection must be made, and the second dose of salt water will suffice. In only two cases has a third injection been necessary. It is remarkable how quickly the urinary suppression will disappear under such treatment.

This treatment is not difficult of execution. The things one must have are (1) a large hollow needle, (2) a piece of rubber tubing, (3) something to act as a funnel. Water that has been filtered is put in a clean pot and boiled, a teaspoonful of common salt being added to each quart, and allowed to cool to 103 degrees. The skin is next cleaned over some convenient superficial vein, and the vein laid bare by an incision of an inch, or inch and a half; it is temporarily covered with a piece of gauze, or perfectly clean cloth. Then the funnel (preferably a small glass one, but it matters not) is attached to one end of the rubber tube, and the needle to the other end. This apparatus is scalded out thoroughly, and then the funnel is filled with the hot, salt solution, and the stream allowed to begin running. While the fluid is still running, the needle is inserted into the vein. The funnel is kept about three feet above the level of the patient, and *must be kept full* by constant pouring, so as not to admit any air, and the needle must be withdrawn while the stream is still flowing. About twelve ounces is the usual amount needed; from eight to sixteen being the rule, according to the size of patient, etc.

This intravenous injection of normal salt solution is thus easily performed, and may be relied upon to give relief if carried out properly at an early period, before the nerve centers become too badly poisoned to revive.

"But," says the timid doctor, "suppose the people will not permit all this, what then?" My only answer is that every true physician, when called to a case of puerpural eclampsia will insist upon having absolute control of the patient, after explaining the gravity of the case. If the friends will not allow this, it is his duty to demand that another physician be immediately sent for, and to remain with the patient, giving chloroform, etc., until the arrival of the second doctor; to explain to him the circumstances, and withdraw from the case. There is no credit to be obtained by lingering. But if the second doctor called be half a man, he will give the friends a good "cussing" (modified to suit the case), insist upon the retention of the first and the adoption of proper treatment, and, together with his brother physician, try to save the lives which have been jeopardized by the delay.

